# Anomalous Case of Hernia

**Published:** 1850-06

**Authors:** Wm. Ring

**Affiliations:** Buffalo


### ART. IV.—*Anomalous case of Hernia.* By Wm. Ring, M. D.

William Bedford, a colored man, came under my observation about the 1st of December, at the Erie county work-house. Age about 60, constitution much impaired by exposure. On examination, I found that he had a strangulated inguinal hernia, about twenty years ago, which was relieved by the ordinary operation, since which time he has worn the common truss. My attention was directed to a tumor on the back and middle part of the thigh, near the size of a large cocoa-nut, with fluctuation on pressure, and moveable, resembling very much an encysted tumor, so far as external appearances were concerned. He complained of pain up and down the thigh, of difficulty in passing water, and general weakness, and these were much aggravated by walking. The tumor made its appearance at the same time as the hernia, was on the same side of the body, and was about as large as a hen's egg. It remained of that size until last fall, when the hernia accidentally became strangulated again, but was relieved by the taxis, and after this the tumor enlarged to the size mentioned above when first examined. He wished me to extirpate it. In consultation with Prof. Hamilton, we concluded to let it remain as it was. From frequent attacks of diarrhœa, he died on the 18th of March, at the poor-house, whither he had been removed a few days before. By the kindness of Dr. John P. Pride, physician to that establishment, I was permitted to make a *post mortem* examination, fifteen hours after death, Dr. Pride being present with me.

On dissection we found that a knuckle of small intestine, a portion of the ileum, had passed out of the cavity of the abdomen, beneath pouparts ligament, over the iliacus internus muscle, thence following the conjoined tendon of the psoas magnus and iliacus internus to the trochanter minor of the os femoris, thence passing under the gluteus maximus directly under the fascia lata of the thigh, where the tumor made its appearance. The bowel, where it passed under pouparts ligament, was on the outside of the femoral vessels, and crural nerve, and two inches from the inguinal hernia outwardly. It passed under the nerve and the femoral artery and vein. The muscular structures were discolored and hardened along the course of the hernia. The intestine had become thickened, and adherent to the adjoining parts, and contracted in its calibre to half an inch, and was filled with the ordinary contents of the bowels. The rectum was also contracted and thickened, and there was a fistula in ano opening on the outside of the anus.

The points of interest in the case which suggest themselves to my mind are:

*First,* Its appearing at the same time as the inguinal hernia.

*Second,* The uncommon situation in which it passed out of the abdomen, — on the outside of the large vessels and nerves of the thigh.

*Third,* The great distance to which the bowel had protruded, — about thirteen inches.

*Fourth,* The great length of time the hernia had existed, and life had been sustained with it in that condition.

WILLIAM RING.

BUFFALO, 1850.

